# Direct valorisation of waste cocoa butter triglycerides via catalytic epoxidation, ring‐opening and polymerisation

**DOI:** 10.1002/jctb.5292

**Published:** 2017-05-24

**Authors:** Dorota D Plaza, Vinzent Strobel, Parminder Kaur KS Heer, Andrew B Sellars, Seng‐Soi Hoong, Andrew J Clark, Alexei A Lapkin

**Affiliations:** ^1^ School of Engineering University of Warwick Coventry UK; ^2^ Department of Chemical Engineering and Biotechnology University of Cambridge UK; ^3^ Aachener Verfahrenstechnik – Process Systems Engineering RWTH Aachen University Aachen Germany; ^4^ Department of Chemistry University of Warwick Coventry UK

**Keywords:** cocoa butter, epoxidation, flow chemistry, food waste, phase‐transfer catalysis, polyurethane, renewable feedstocks, ring‐opening, polymerisation

## Abstract

**BACKGROUND:**

Development of circular economy requires significant advances in the technologies for valorisation of waste, as waste becomes new feedstock. Food waste is a particularly important feedstock, containing large variation of complex chemical functionality. Although most food waste sources are complex mixtures, waste from food processing, no longer suitable for the human food chain, may also represent relatively clean materials. One such material requiring valorisation is cocoa butter.

**RESULTS:**

Epoxidation of a triglyceride from a food waste source, processing waste cocoa butter, into the corresponding triglyceride epoxide was carried out using a modified Ishii‐Venturello catalyst in batch and continuous flow reactors. The batch reactor achieved higher yields due to the significant decomposition of hydrogen peroxide in the laminar flow tubular reactor. Integral and differential models describing the reaction and the phase transfer kinetics were developed for the epoxidation of cocoa butter and the model parameters were estimated. Ring‐opening of the epoxidised cocoa butter was undertaken to provide polyols of varying molecular weight (M_w_ = 2000–84 000 Da), hydroxyl value (27–60 mg KOH g^−1^) and acid value (1–173 mg KOH g^−1^), using either aqueous ortho‐phosphoric acid (H_**3**_PO_**4**_ **)** or boron trifluoride diethyl etherate (BF_**3**_ **·**OEt_2_)‐mediated oligomerisation in bulk, using hexane or tetrahydrofuran (THF) as solvents. The thermal and tensile properties of the polyurethanes obtained from the reaction of these polyols with 4,4′‐methylene diphenyl diisocyanate (MDI) are described.

**CONCLUSION:**

The paper presents a complete valorisation scheme for a food manufacturing industry waste stream, starting from the initial chemical transformation, developing a process model for the design of a scaled‐up process, and leading to synthesis of the final product, in this case a polymer. This work describes aspects of optimisation of the conversion route, focusing on clean synthesis and also demonstrates the interdisciplinary nature of the development projects, requiring input from different areas of chemistry, process modelling and process design. © 2017 The Authors. Journal of Chemical Technology & Biotechnology published by John Wiley & Sons Ltd on behalf of Society of Chemical Industry.

**NOTATION**
a: interfacial area (m^2^/m^3^)
c: concentration (mol/l)
$\bar{c}$: mean concentration within a droplet (mol/l)
$\overset{=}{c}$: mean concentration within a phase (mol/l)
D: diffusion coefficient (m^2^/s)
E_a_: activation energy (J/mol)
H: partition coefficient
ΔH_r_: enthalpy of the reaction (J/mol)
k: reaction rate constant in Eqs. (5) & (6) (l/mol s), mass transfer coefficient in Eq. (23) (m/s)
K: overall mass transfer coefficient (m/s)
n_i , 0_: initial amount of reagent (mol)
${\dot{q}}_{\text{dos}}$: heat flow rate connected with dosing of reagents (W)
${\dot{q}}_{\text{lid}}$: heat flow rate through the reactor cap (W)
${\dot{q}}_{\text{mix}}$: heat flow rate of mixing different enthalpies of the reacted substances (W)
${\dot{q}}_{\text{phase}}$: heat flow rate of phases which are changed during the reaction (W)
${\dot{q}}_{\text{react}}$: reaction heat flow rate (W)
${\dot{q}}_{\text{stirr}}$: heat flow rate of stirring of reagents (W)
${\dot{q}}_{\text{true flow}}$: true heat flow (W)
r: reaction rate (mol/l s)
R: droplet radius (m)
R_g_: universal gas constant (J/mol K)
t: time (s)/(min)
T: temperature (K)
V: volume (l)
w: weighting factor

**Subscripts**
aq: aqueous phase
cat: inactive state of catalyst
$\bar{\text{cat}}$: active state of catalyst
CB: cocoa butter
epx: epoxide product
k: element of a droplet size distribution
org: organic phase
ref: reference value for re‐parameterization for Arrhenius equation

**Greek letters**
ξ: dimensionless radial coordinate

## INTRODUCTION

**Scheme 1:**
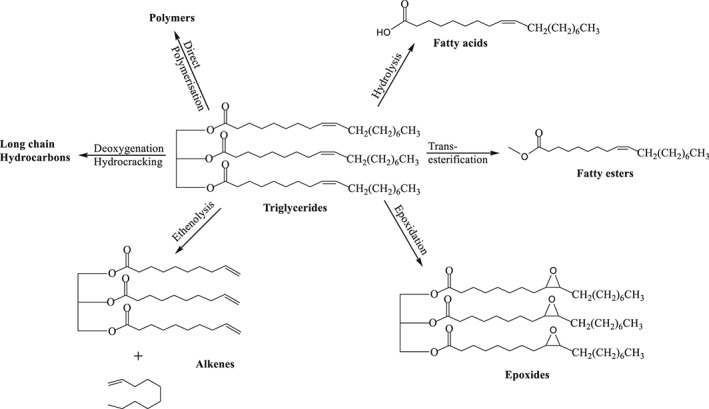
Triglycerides conversion routes.

Food waste represents a particularly attractive source of complex molecular structures for intermediate and high‐value chemical products due to its low cost, close proximity to chemical and biotechnology industrial hubs, and societal pressure on its more complete utilisation. Better utilisation of food waste would contribute significantly to the vision of the circular economy advocated, among many, by the European Commission.^1^ Within the range of molecules of interest based on bio‐feedstocks including food waste, triglycerides form one of the largest classes (by volume) presently used. Triglycerides are composed of three fatty acids joined at a glycerol juncture. They have been transformed into a variety of products, for example for transportation fuels, food, cosmetics, detergents, lubricants directly or indirectly.^2^ The various conversion routes of triglycerides are shown in Scheme 1.

Conversion of triglycerides into biodiesel (fatty esters) is one of the most useful ways of utilising their intrinsic calorific value.^3^ These fatty esters are also used for the production of terminal olefins and epoxides via metathesis/ethenolysis and epoxidation, respectively, which are then further used as intermediates in chemical reactions or as new polymer precursors.^4^, ^5^ Hydrolysis of triglycerides produces fatty acids, in addition to glycerol, which are major components and precursors for products such as soaps, detergents, fatty alcohols, cosmetics, pharmaceuticals, and food. Triglycerides have also been extensively utilised to produce polymeric materials.

The first approach for the preparation of polymers from triglycerides is the direct polymerisation of the double bonds or other reactive functional groups present in the fatty acid chain.^6^ However, this is difficult due to the lack of active functional groups. Hence, triglycerides can be functionalised or chemically transformed into other products, which in turn can be used as monomers for polymerisation or chemical intermediates.^6^, ^7^

Deoxygenation and hydrocracking of vegetable oils or triglycerides for the production of long chain hydrocarbons has been studied extensively.^8^, ^9^, ^10^, ^11^ Selective conversion of triglycerides to olefins is also considered as a potential substitute to petroleum‐based feedstocks as starting materials for speciality chemicals^12^ in the strategy we would call ‘CO_2_ avoidance’. Metathesis/ethenolysis of vegetable oils or triglyceride has been reported for the production of olefins which would be useful in the synthesis of polymers as well as intermediates for other products.^13^, ^14^, ^15^

Epoxidation is another important reaction leading to reactive intermediates for production of polymers, emulsifiers and lubricants.^16^, ^17^, ^18^ Batch syntheses of fatty epoxides are well studied from free fatty acids and fatty esters, and some are exploited commercially. Epoxidation of triglycerides, on the other hand, is less studied^19^, ^20^, ^21^, ^22^, ^23^, ^24^, ^25^ since this reaction is: (a) technically more challenging; and (b) the product scope is not well explored. However, direct epoxidation of triglycerides, if technically feasible, may be a useful pathway for the production of novel functional materials, avoiding the need for transesterification and, hence, using more of the carbon atoms of the original feedstock in the products.

Epoxidation of soybean oils was investigated in a series of papers.^26^, ^27^ Conventional batch epoxidation with hydrogen peroxide in the presence of acetic or formic acid and mineral acid was reported with a yield of up to 75% at temperatures between 60–80°C with a typical batch time of 5 h. A decrease in reaction time and slight improvement of the overall energy efficiency was possible if the reaction temperature was increased and the reaction was performed in a microreactor with efficient heat transfer. However, at temperatures above 150°C decomposition of hydrogen peroxide dominates and the reaction yield is significantly reduced compared with the batch, lower‐temperature, process. A more recent study reported a lower temperature epoxidation of sunflower oil in an unstable two‐phase flow regime using surfactant‐less biphasic H_2_O_2_ epoxidation catalysed by a W‐based system.^28^ The reaction system was not optimised for high per‐pass conversion, but the ease of phase separation after the reaction allowed simple re‐circulation to reach higher conversion in a loop‐reactor mode.

Epoxidation of longer‐chain olefins suffers from severe mass transfer limitations due to high viscosity, and represents significant safety hazard if run as a large‐scale batch process. Continuous flow processing may have considerable advantages in the production of epoxides compared with batch conditions if the benefits of enhanced mass and heat transfer, typically reported for flow processes,^29^ could be exploited in the case of a high‐viscosity reaction medium.

There are an increasing number of literature examples of epoxidations of longer olefins being carried out under flow conditions. Epoxidations of 1,5‐cyclooctadiene, 1,2‐dihydronaphthalene, ethyl *trans*‐3‐hexenoate, dodec‐1‐ene, duroquinone, *trans*‐stilbene, *cis*‐stilbene, ethyl trans‐cinnamate, methyl *trans*‐2‐methyl‐2‐pentenoate using homogeneous stoichiometric reactant HOF·MeCN was performed with high yield under continuous flow.^30^ Epoxidations of cyclohexene, trans‐3‐heptene, cis‐3‐heptene, 3‐methyl‐2‐pentene, bicyclo[2.2.1]hept‐2‐ene, 1‐hexene, methyl 2‐cyclohexenylcarboxylate, trans‐2‐hexenyl acetate, 2‐cyclohexenylacetate, trans‐2‐hexenyl acetate, 5‐hexenyl acetate were performed using 2‐percarboxyethyl‐functionalised silica in supercritical carbon dioxide at a pressure of 250 bar and temperature 40°C in flow, reporting products yields of > 99%.^31^ Side reactions to acid catalysed ring opening or other side products were suppressed due to using an anhydrous supported peracid. Epoxidation of cyclohexene with the *in situ* generated *m*‐chloroperbenzoic acid was recently reported with up to 95% yield of the oxide under optimised conditions.^32^ Epoxidation of styrene with the same reagent in the presence of excess methylmorpholine‐N‐oxide in a microfluidic reactor coated with a polymer‐immobilised Mn (III)‐salen catalyst was reported with a 190‐fold increase in the space time yield compared with a batch recipe.^33^ The improvement was attributed to a better contact between reactants and the catalyst. Epoxidation of cyclooctene using a sacrificial aldehyde reactant in the Mukaiyama epoxidation was performed in a segmented continuous flow microreactor, reporting 100% selectivity to the epoxide at a short residence time of 2 min.^34^ However, there are only two examples of epoxidation of triglycerides under flow conditions, the epoxidation of soybean oil^26^, ^27^ and of sunflower oil.^28^

**Scheme 2:**
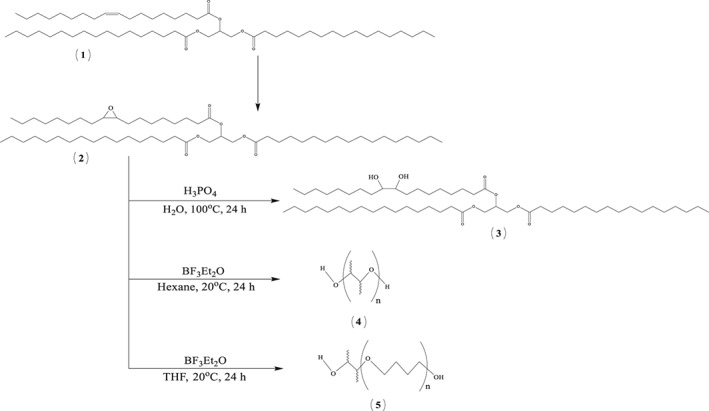
Reaction scheme studied in the present work. (**1**) Cocoa butter, (**2**) the epoxidised cocoa butter, (**3**) ring‐opened cocoa butter from the reaction with aqueous H_3_PO_4_ at 100°C, (**4**) ring‐opened cocoa butter from reaction with BF_3_Et_2_O in hexane at 20°C, (**5**) ring‐opened cocoa butter from the reaction with BF_3_·Et_2_O in THF at 20°C.

Here we report investigation of epoxidation and further synthesis of polymers starting from cocoa butter, which is a post‐manufacture waste product from the food industry. Recently, we reported metathesis of cocoa butter triglyceride in batch and flow conditions to produce 1‐decene.^35^ In the present paper, we first report epoxidation of cocoa butter with hydrogen peroxide catalysed by an Ishii‐Venturello catalyst,^36^ to give its epoxide in batch and flow conditions. Literature reports epoxidation performed as a phase transfer catalytic reaction with quaternary ammonium or phosphonium salts as phase transfer reagents.^36^, ^37^ Earlier papers also report similar epoxidations performed in chlorinated solvents.^36^, ^37^, ^38^, ^39^, ^40^, ^41^ However, the reaction can be performed solventless as long as it is properly emulsified.^20^, ^42^ Significant drawbacks of these reactions are the decomposition of hydrogen peroxide and the acid‐catalysed side reactions to ring opening of epoxides, reducing the product yield. A modified Ishii‐Venturello catalyst was reported earlier^43^, ^44^, ^45^ with tungsten powder being used to obtain the active form of the catalyst *in situ*. The modified catalytic system is characterised by high selectivity and conversion towards the epoxidation reactions. Here, a detailed kinetic model was developed to describe the reaction and phase transfer; the kinetic parameters were evaluated. Conversion of epoxides to the corresponding polyols is reported. Polyols of varying molecular weight and hydroxyl value were prepared from the epoxidised cocoa butter. Finally, polymerisation of these polyols, used as monomers, to prepare polyurethanes as well as thermal and tensile properties of polyurethanes are described. The present work describes the complete process of conversion of waste cocoa butter into useful polymeric materials which is otherwise destined for landfill, Scheme 2.

## EXPERIMENTAL

### Materials

Cocoa butter raw material was obtained from Whitland Ltd. It is a by‐product of chocolate manufacturing process, which cannot be re‐used for foods manufacture. Tungsten powder (12 µm, 99.9%), Adogen 464 and deuterated chloroform were purchased from Aldrich. Ortho‐phosphoric acid (85% wt. in H_2_O), sodium chloride, chloroform, hydrogen peroxide (30% wt. in H_2_O) were purchased from Fisher Chemicals. Anhydrous magnesium sulphate was received from Acros Organics. Reagents were used without further purification. Diethyl ether, hexane, boron trifluoride diethyl etherate (BF_3_·Et_2_O), sodium bicarbonate, tetrahydrofuran (THF), methylene diphenyl diisocyanate (MDI) were purchased from Sigma‐Aldrich.

### Batch epoxidation

In a typical experiment, tungsten powder (0.081 g, 0.44 mmol), hydrogen peroxide (1.2 mL) and deionised water (0.6 mL) were introduced into a 20 mL round‐bottom flask with a stirrer bar. The mixture was heated to 50°C and stirred to dissolve the tungsten powder for about 45 min. Cocoa butter (15 g, 17.3 mmol) and Adogen 464 (0.105 g, 0.26 mmol) were placed into a 250 mL round‐bottom‐flask, melted and stirred at a set reaction temperature: 50, 60 or 80°C. The mixture containing the dissolved tungsten catalyst was cooled, then ortho‐phosphoric acid (85 wt%, 0.081 g, 0.703 mmol) in 2 mL of deionised water was added under continuous stirring. The solution of the catalyst, hydrogen peroxide (2.88 mL) and deionised water (22.9 mL) were added to the molten cocoa butter. The mixture was stirred and maintained at the desired reaction temperature.

Four different catalyst compositions were used, see Table 1. On the completion of reaction, the resulting emulsion was cooled and saturated sodium chloride (25 mL) and chloroform (25 mL) were added. The organic phase was separated and washed with saturated sodium chloride again (25 mL). The organic layer was dried over anhydrous magnesium sulphate and after filtration the solvent was removed using rotary evaporator.

**Table 1:** Molar equivalents of the catalyst components used in the epoxidation of cocoa butter

Reaction number	W	H_2_O_2_	H_3_PO_4_	H_2_O
1	0.63	57	1	2267
2	0.63	39	1	2190
3	0.63	25	1	2129
4	0.54	45	1	2177

### Continuous flow epoxidation

**Figure 1:**
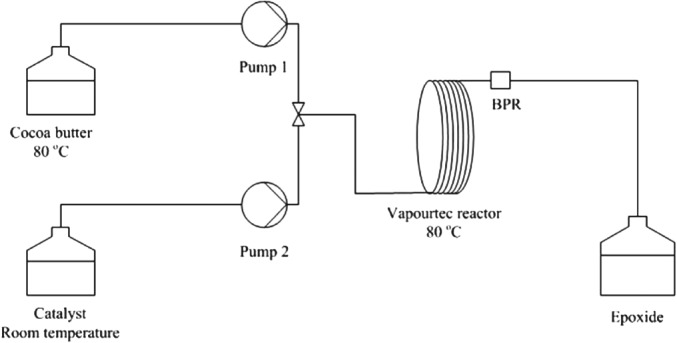
Schematic diagram of the reactor configuration for epoxidation of cocoa butter under flow conditions.

Epoxidation of cocoa butter was performed using a Vapourtec R Series reaction system. The Vapourtec module was configured as shown in Figure 1. In the flow experiments viscosity of cocoa butter was reduced by adding 10 mL of toluene to the feed flask, containing cocoa butter (0.081 g, 17.3 mmol) and Adogen 464 surfactant (0.105 g, 0.26 mmol). This mixture was maintained at 80°C. Catalyst composition corresponds to the reaction 1 in Table 1, with the following molar ratios of all reactants with respect to the average amount of double bonds in cocoa butter (1.15 mol of double bonds per 1 mol of cocoa butter): cocoa butter/W/H_2_O_2_ / H_2_O / H_3_PO_4_ = 1 / 0.025 / 7.86 / 81.91 / 0.040. Reaction was performed in a 10 mL tubular reaction module (PTFE, 1 mm ID and 12.7 m length) at 80°C.

### Reaction calorimetry

The determination of the enthalpy of epoxidation of cocoa butter was carried out in the isothermal mode using a reaction calorimeter (Chemisens, CPA‐202). The reaction calorimeter was first loaded with cocoa butter (15.0 g, 17.3 mmol) and Adogen 464 (0.105 g, 0.26 mmol). The mixture was heated to 80°C and stirred at 500 rpm until the true heat flow showed stable baseline. Then the solution of catalyst was kept at 24.7°C and prepared according to the standard procedure for epoxidation and was injected into the reactor.

The energy balance for reaction calorimetry by CPA 202 reactor can be written as follows:

(1) $${\dot{q}}_{\text{true flow}}+{\dot{q}}_{\text{lid}}={\dot{q}}_{\text{react}}+{\dot{q}}_{\text{mix}}+{\dot{q}}_{\text{phase}}+{\dot{q}}_{\text{dos}}+{\dot{q}}_{\text{stirr}}$$

Where the terms correspond to heat flow through lid, due to reaction, mixing, phase change, dosing and stirring.

The enthalpy of the reaction was calculated as shown in Equation (2):^46^, ^47^

(2) $$\Delta H_{r}=\frac{\int_{0}^{\infty }{\dot{q}}_{\text{true flow}}\text{dt}}{n_{i,0}}$$

### Kinetic model development

The kinetic model was developed for the batch epoxidation. This is a liquid–liquid two‐phase reaction. The organic phase is pure molten cocoa butter, and the aqueous phase is aqueous hydrogen peroxide solution with the catalyst. According to ref 41 a precursor with the structure [PW_12_O_40_]^3−^ forms in situ from tungsten powder, hydrogen peroxide and ortho‐phosphoric acid in an aqueous solution. The active polyperoxotungstate species {PO_4_[WO(O_2_)_2_]_4_}^3−^ is formed by subsequent oxidation with hydrogen peroxide. This anionic active species binds to the cationic phase transfer agent (PTA), typically a quaternary ammonium salt, and is transferred into the organic phase where it catalyses the epoxidation of a double bond. The inactive catalyst transfers back into the aqueous phase, where it is reactivated by oxidation with hydrogen peroxide and the cycle starts again. While this seems like a clear linear process, this mechanism is not fully understood. The exact catalytic species and how many different tungsten species are formed is not known. It is also not understood, whether the phase transfer agent permanently binds to the tungstic catalyst to form a PTA–catalyst complex or only reversibly attaches for the phase transfer step. In this work it is assumed that the PTA and the tungsten catalyst bond permanently to form a single PTA–catalyst species and, therefore, polyperoxotungstate catalyst and the PTA are not modelled as separate components, but assumed to form a single PTA–catalyst whose overall amount is constant during the experiment and can change between an active and passive state in each phase.

Both reactions, the epoxidation and catalyst activation, are assumed to occur only within the well‐mixed parts of both the phases. Between each of the reaction steps a phase transfer has to take place. Hence, reaction kinetics and mass transfer are directly coupled within the reaction mechanism. It is further assumed that the organic and the aqueous phases are completely immiscible and the active and passive catalyst species are the only components which transfer between the phases. The interfacial area has no direct effect on the rate. The stoichiometry of both the reactions is given in Equations (3) and (4):

Aqueous phase: $\text{cat}+H_{2}O_{2}\to \bar{\text{cat}}+H_{2}O$ (3)

Organic phase: $\text{CB}+\bar{\text{cat}}\to \text{epx}+\text{cat}$ (4)

The kinetic rate equations were written following the re‐parameterisation of the Arrhenius equation as shown below so as to improve the identifiability of the parameters. The experiments were assumed to occur under isothermal conditions (the assumption was validated with the heat of reaction measurements and, therefore, isothermal reference rate constants were used; see Supporting information).

Aqueous phase: $r_{\text{aq}}=k_{\text{ref},\text{aq}}\text{exp}\left(\frac{E_{a,\text{aq}}}{R_{g}}\left[\frac{1}{T_{\text{aq}}}-\frac{1}{T_{\text{ref}}}\right]\right)c_{\text{cat},\text{aq}}c_{H2O2}$ (5)

Organic phase: $r_{\text{org}}=k_{\mathrm{ref},\mathrm{org}}\text{exp}\left(\frac{E_{a,\text{org}}}{R_{g}}\left[\frac{1}{T_{\text{org}}}-\frac{1}{T_{\text{ref}}}\right]\right)c_{\bar{\text{cat}},\text{org}}c_{\text{CB}}$ (6)

**Figure 2:**
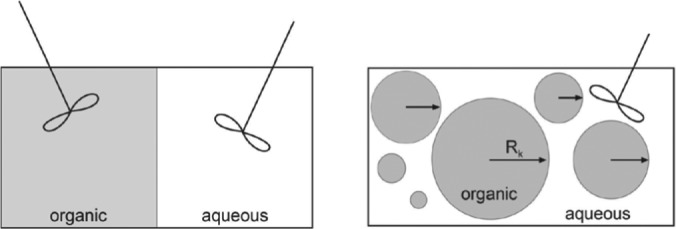
Schematic representation of an integral model (A. left) and a differential model (B. right).

The kinetic rates were combined with the mass transfer steps to derive a complete model for the epoxidation. Two different modelling approaches were used: (1) an integral model, which assumes two ideally mixed phases and an overall mass transfer coefficient, Figure 2(A); and (2) a semi‐differential model, which potentially yields more realistic results, but leads to significant numerical complexity due to the increased number of parameters and the need to fit an integral of the droplet concentration to the measurement data, Figure 2(B).

#### *Integral model*

The integral model assumes perfectly mixed phases with uniform concentrations and temperatures within each phase. Interfacial mass transport occurs across an interfacial area and is approximated by an overall mass transport coefficient. The volume of each phase is known from the experimental data and can be assumed constant, since the molar volume of cocoa butter does not change significantly with epoxidation and the aqueous phase is mostly water and will not undergo significant volume changes. Therefore, component mass balances were formulated in terms of concentrations. Since it has been assumed that cocoa butter and the product epoxide do not diffuse into the aqueous phase, their mass balances are solely dependent on the reaction rates. To reduce the complexity of the model, the same mass transfer coefficient is used for both catalyst species.

Organic phase:

(7) $$\frac{{\text{dc}}_{\bar{\text{cat}},\text{org}}}{\text{dt}}=\text{Ka}\left(c_{\bar{\text{cat}},\text{org}}-H_{\bar{\text{cat}}}c_{\bar{\text{cat}},\text{aq}}\right)-r_{\text{org}}$$

(8) $$\frac{{\text{dc}}_{\text{cat},\text{org}}}{\text{dt}}=\text{Ka}\left(c_{\text{cat},\text{org}}-H_{\text{cat}}c_{\text{cat},\text{aq}}\right)-r_{\text{org}}$$

(9) $$\frac{{\text{dc}}_{\text{CB}}}{\text{dt}}=-r_{\text{org}}$$

(10) $$\frac{{\mathrm{dc}}_{\mathrm{epx}}}{\mathrm{dt}}={\text{r}}_{\text{org}}$$

The mass balances for the aqueous phase are formulated in the same way. However, the phase volume ratio is needed to correct the interfacial flux and the opposite sign is needed. Water and hydrogen peroxide are assumed to remain completely within the aqueous phase.

Aqueous phase:

(11) $$\frac{{\text{dc}}_{\bar{\text{cat}},\text{aq}}}{\text{dt}}=\text{Ka}\frac{V_{\text{org}}}{V_{\text{aq}}}\left(H_{\bar{\text{cat}}}c_{\bar{\text{cat}},\text{aq}}-c_{\bar{\text{cat}},\text{org}}\right)+r_{\text{aq}}$$

(12) $$\frac{{\text{dc}}_{\text{cat},\mathrm{aq}}}{\mathrm{dt}}=\mathrm{Ka}\frac{V_{\text{org}}}{V_{\mathrm{aq}}}\left(H_{\text{cat}}c_{\text{cat},\mathrm{aq}}-c_{\mathrm{cat},\mathrm{org}}\right)-r_{\mathrm{aq}}$$

(13) $$\frac{{\mathrm{dc}}_{H2O2}}{\mathrm{dt}}=-r_{\mathrm{aq}}$$

(14) $$\frac{{\mathrm{dc}}_{H2O}}{\mathrm{dt}}=r_{\mathrm{aq}}$$

Equations (7)–(14) compose the integral model, which was implemented in gPROMS model builder, and used for parameter estimation. The reliability of the parameter estimation was checked by two statistical tests, t‐test and χ^2^ test, and the confidence ellipsoid. The t‐test established the statistical significance of each parameter. The χ^2^ test related to the goodness of the model fit is satisfied when the weighted residual value is less than the χ^2^ value. The confidence ellipsoid takes into account the correlation between the estimates of a kinetic constant at a reference temperature and the activation energy for each reaction as well as the mass transfer correlation parameters.

#### *Differential model*

The integral model, although widely used in literature, does not reflect the real physical situation. A logical step towards a more realistic representation of the physical situation is the assumption of spherical droplets where the only mass transport inside the droplet is radial diffusion. The differential model can be applied to many droplets with different droplets sizes, implying a droplet size distribution.

For the differential model, the equations for the organic phase are replaced with one‐dimensional partial differential equations for radial diffusion inside a sphere. There are no mass transport terms in the component mass balances because the interfacial mass transport will be accounted for by the boundary conditions. For the aqueous phase the equations stay nearly the same. Only the overall mass transfer coefficient is replaced by the aqueous side mass transfer coefficient. The differential equations were written as dimensionless, ranging from 0 to 1, and allowing the droplet radius to be multiplied as an additional variable. With this formulation different droplet radii can be implemented in the model, enabling the simulation of a droplet size distribution. A possible distribution can be formulated as a vector *‘R’* containing different radii, which are weighted by additional weight constants *‘w’*.

(15) $$R=\left[R_{1},R_{2},\ldots ,R_{k},\ldots R_{m}\right]$$

(16) $$w=\left[w_{1},w_{2},\ldots ,w_{k},\ldots w_{m}\right]$$

(17) $$\sum_{k=1}^{m}w_{k}=1$$

For a given droplet radius, the mass balances for the organic phase can be written as partial differential equation in spherical coordinates, with integration limits for *ξ* from 0 to 1. It should be noted that the rate equation, as a function of concentration, is now also dependent on the droplet radii:

(18) $$\frac{\partial c_{\bar{\mathrm{cat}},\mathrm{org}}}{\partial t}=\frac{1}{R_{k}^{2}}\frac{D_{\bar{\mathrm{cat}}}}{\xi^{2}}\frac{\partial }{\partial \xi }\left[\xi^{2}\frac{\partial c_{\bar{\mathrm{cat}},\mathrm{org}}}{\partial \xi }\right]-r_{\mathrm{org}}\left(\xi \right)$$

(19) $$\frac{\partial c_{\mathrm{cat},\mathrm{org}}}{\partial t}=\frac{1}{R_{k}^{2}}\frac{D_{\mathrm{cat}}}{\xi^{2}}\frac{\partial }{\partial \xi }\left[\xi^{2}\frac{\partial c_{\mathrm{cat},\mathrm{org}}}{\partial \xi }\right]+r_{\mathrm{org}}\left(\xi \right)$$

(20) $$\frac{\partial c_{\mathrm{CB}}}{\partial t}=\frac{1}{R_{k}^{2}}\frac{D_{\mathrm{CB}}}{\xi^{2}}\frac{\partial }{\partial \xi }\left[\xi^{2}\frac{\partial c_{\mathrm{CB}}}{\partial \xi }\right]-r_{\mathrm{org}}\left(\xi \right)$$

(21) $$\frac{\partial c_{\mathrm{epx}}}{\partial t}=\frac{1}{R_{k}^{2}}\frac{D_{\mathrm{epx}}}{\xi^{2}}\frac{\partial }{\partial \xi }\left[\xi^{2}\frac{\partial c_{\mathrm{epx}}}{\partial \xi }\right]+r_{\mathrm{org}}\left(\xi \right)$$

Additional to initial conditions, which have to be defined from *ξ*=0 to *ξ*=1, boundary conditions for the spatial gradients are needed. At the centre of the droplet all gradients equal zero:

(22) $${\left.\frac{\partial c}{\partial \xi }\right|}_{\xi =0}=0$$

The boundary condition at the droplet surface for *ξ*=1 was derived from a surface balance and takes the following form for $\bar{\mathrm{cat}}$ and *cat*:

(23) $${\left.\frac{\partial c_{i,\text{org}}}{\partial \xi }\right|}_{\xi =1}=\frac{Rk_{\mathrm{aq}}}{D_{i}}\left(H_{i}c_{i,\mathrm{aq}}-c_{i,\mathrm{org}}\left(1\right)\right)$$

The aqueous side mass transfer resistance *k*_*aq*_ replaces the overall mass transfer coefficient *K*, because the mass transfer resistance in the organic phase is now described by the diffusion coefficient in Equations (18)–(21).

If the model is fitted to experimental data, the available measurements will correlate with the average concentration within either phase. Thus, for the organic phase the mean of all average concentrations of all different droplet sizes has to be calculated. Within a single droplet the average concentration can be calculated by integration over the radius:

(24) $${\bar{c}}_{i,\text{org}}\left(R_{k}\right)=3\int_{0}^{1}c_{i,\text{org}}\left(\xi \right)\xi^{2}d\xi ,i\in \left\{\bar{\text{cat}},\text{cat},\text{CB},\text{epx}\right\}$$

Consequently, the arithmetic mean of all integrated values yields the overall mean concentration of a species in the organic phase:

(25) $${\overset{=}{c}}_{i,\mathrm{org}}=\sum_{i=1}^{m}w_{k}{\bar{c}}_{i,\mathrm{org}}\left(R_{k}\right)$$

The model was implemented in gPROMS and was used for simulation, however, no parameter estimation could be performed with the differential model: only one radius was taken and monodispersed droplets were assumed.

### Ring opening of the epoxidised cocoa butter

Ring opening was carried out by three approaches to furnish polyols with different molecular weights and hydroxyl numbers.

#### *Ring opening using H_3_PO_4_*

The epoxidised cocoa butter (5 g, 5.7 mmol, 1.0 equiv.) and water (5.7 mL, 1 mol L^−1^) were heated to 100°C. Adogen 464 (0.012 g, 2.5% wt.) was added followed by H_3_PO_4_ (0.56 g, 5.7 mmol, 1.0 equiv). The reaction mixture was heated at 100°C for 24 h. The reaction was extracted with diethyl ether, washed with water (2 × 10 mL) followed by saturated NaCl (10 mL). The organic layer was dried over MgSO_4_ and the solvent removed *in vacuo* to give a pure product (97%, Polyol (**3**), Scheme 2).

#### *Ring opening using BF_3_.Et_2_O in hexane*

The epoxidised cocoa butter (50.0 g, 57 mmol) was weighed into a round bottom flask and hexane (80 mL) was added to it. The reaction mixture was stirred and heated at 20°C under N_2_ atmosphere for 30 mins before BF_3_·Et_2_O (0.8 g, 5.7 mmol) was added drop‐wise to the mixture. After the addition of BF_3_·Et_2_O was completed, the reaction temperature was maintained at 20°C for another 24 h. The reaction mixture was then poured into a separation funnel and washed with sodium bicarbonate solution (250 mL). The aqueous layer was separated from the organic layer and CHCl_3_ (500 mL) was added to the organic layer. The organic layer was dried over anhydrous MgSO_4_ and CHCl_3_ was removed *in vacuo* to give a yellowish liquid (49.3 g, Polyol (**4**), Scheme 2).

#### *Ring opening using BF_3_.Et_2_O in THF*

The epoxidised cocoa butter (50 g, 57 mmol) was weighed into a round bottom flask and dry THF (250 mL) was added. The reaction mixture was stirred and heated at 20°C under N_2_ atmosphere for 30 mins. Then BF_3_.Et_2_O (0.8 g, 5.7 mmol) was added drop‐wise to the mixture. After the addition of BF_3_.Et_2_O was complete, the reaction temperature was maintained at 20°C under a N_2_ atmosphere for another 24 h. Then, the reaction mixture was poured into a separation funnel and saturated NaCl solution (200 mL) and CHCl_3_ (300 mL) were added to the reaction mixture. The aqueous layer was separated from the organic layer and the organic layer was neutralized with saturated sodium bicarbonate solution (100 mL). The organic layer was again washed with NaCl solution (200 mL). The organic layer was dried over anhydrous MgSO_4_ followed by removal of solvent *in vacuo* to yield a clear viscous liquid (120 g, Polyol (**5**), Scheme 2).

### Polymerisation of polyols to polyurethanes (PU)

The monomers **3–5** of Scheme 2 (1.0 eq) were heated in a 50 mL round bottomed flask to 60°C under vacuum for 1 h to remove air and residual solvent. MDI (1.05 eq) was added and the vacuum was reapplied for 2 min at 60°C with simultaneous gentle stirring to prevent air entrapment in the polymer. The monomers were then cast into a mould and cured at 60°C in an oven overnight.

### Characterisation

#### *Cocoa butter*

Fatty acid composition was determined by gas chromatography. The GPC analysis of cocoa butter was performed using a PL‐GPC 50 integrated with PL‐BV 400RT refractive index detector and a rheometer. THF was used as the eluent at a flow rate of 1.0 mL min^−1^. The calibration curve for GPC analysis was obtained with poly(methyl methacrylate) (PMMA) standards.

#### *Epoxidation*

TLC was carried out using Merck silica gel coated aluminium sheets as the stationary phase (Merck Kieselgel 60F_254_ 230–400 mesh). The TLC plate was visualised using a UV lamp (254 nm) and stained using potassium permanganate solution. ^1^H and ^13^C NMR were performed on a Bruker DPX‐400 spectrometer, at 400 MHz and 100 MHz, respectively. All chemical shifts were in parts per million (ppm) relative to the tetramethylsilane (TMS) internal standard (0.03 % v/v, 0.00 ppm). Coupling constants (*J*) were expressed in Hertz (Hz). ^1^H NMRs were obtained at room temperature and using deuterated chloroform as solvent. The progress of epoxidation of cocoa butter was monitored by disappearance of double bond (δ = 5.30–5.39 ppm) and appearance of oxirane bond (δ = 2.85–2.96 ppm). Glycerol bond signal at 4.25–4.34 ppm was used as an internal standard, according to methodology described elsewhere.^48^ Infrared spectra were recorded on a Bruker ALPHA platinum ATR Fourier transform spectrometer. Absorptions were recorded in wavenumbers (cm^−1^). Mass spectrometry was achieved using an Agilent 6130B single Quad (ESI).

#### *Ring opening*

^1^H and ^13^C NMR were performed on a Bruker DPX‐400 spectrometer, at 400 MHz and 100 MHz, respectively. All chemical shifts were in parts per million (ppm) relative to the tetramethylsilane (TMS) internal standard (0.03% v/v, 0.00 ppm). Coupling constants (*J*) were expressed in Hertz (Hz). Infrared spectra were recorded on a Bruker ALPHA platinum ATR Fourier transform spectrometer. Absorptions were recorded in wavenumbers (cm^−1^). Mass spectrometry was achieved using an Agilent 6130B single Quad (ESI). GPC was performed on an Agilent 390‐MDS with autosampler using a PL gel 5.0 µm bead‐size guard column (50 × 7.5 mm), followed by two linear 5.0 µm bead‐size PL gel Mixed D columns (300 × 7.5 mm) and a differential refractive index detector. Using CHCl_3_ as eluent the system was calibrated using linear poly(styrene) Easi Vial standards (Agilent Ltd) range from 162 to 5×10^5^ Da. Data were collected and analysed using Cirrus GPC/SEC (v. 3.3) and Agilent GPC/SEC software. TGA, DSC and melting points were carried out using Metler Toledo DSC1‐Star with 40 µL standard aluminium pans and autosampler. Samples heated from −100–70°C, cooled to −100 and heated from −100–600°C under N_2_ atmosphere for DSC and heated from 25–600°C for TGA.

#### *Polymerisation*

TGA, DSC and melting points were carried out using Metler Toledo DSC1‐Star with 40 µL standard aluminium pans and autosampler. Samples were heated from −100–70°C, cooled to −100°C and heated from −100–600°C under N_2_ atmosphere for DSC and heated from 25–600°C for TGA.

## RESULTS AND DISCUSSION

### Epoxidation

#### *Characterisation of cocoa butter*

Cocoa butter is primarily composed of triglycerides, of which there are a variety of combinations of glycerol with palmitic (P), stearic (S) and oleic (O) acids. Other acid residues are present in lower quantities (palmitoleic acid, myristic acid, arachidic acid, and linoleic acid). The ratios of triglycerides vary as a response to numerous effects, major factors being the geographical source of the bean and the time of the year at harvest, typically variations are within 1–4%. Of the six major triglycerides present (16 have been identified), all contain oleic or linoleic unsaturated acid residues. For the particular sample of cocoa butter used in this study, the fatty acid composition as determined by GC is shown in Table 2, together with the fraction of total double bonds represented by each component. As we have not studied samples of cocoa butter with different compositions we cannot comment in the paper on variation in the epoxidation performance due to variation in the composition.

**Table 2:** Composition of cocoa butter

Fatty acids	Fatty acids in CB (%)	Double bonds in CB (%)
Palmitic (C16:00)	25.8	0
Palmitoleic (C16:1n7)	0.3	0.8
Stearic (C18:00)	37.9	0
Oleic (C18:1n9c)	32.2	84.1
Linoleic (C18:2n6c)	2.9	15.1
Arachidic (C20:00)	0.9	0

#### *Batch epoxidation*

**Figure 3:**
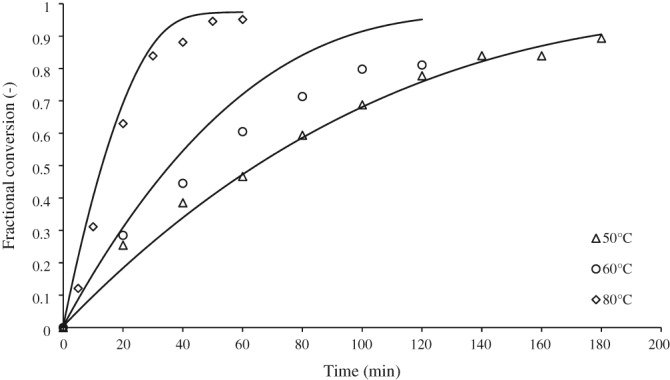
The dependence of conversion of cocoa butter on temperature in batch epoxidations. Symbols represent experimental data; lines represent the integral model fit.

**Figure 4:**
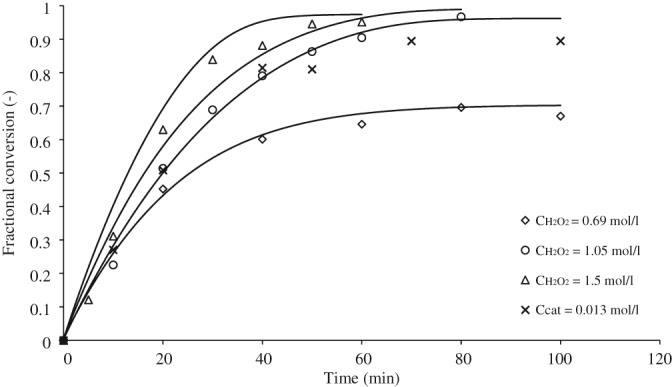
The effect of catalyst composition on conversion at 80°C. The reaction numbers correspond to catalyst compositions shown in Table 1. 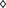 reaction (1), 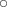 reaction (2), 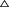 reaction (3), 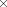 reaction (4). Lines represent the integral model fit.

Epoxidation of cocoa butter was performed at different temperatures, catalyst concentrations and initial concentrations of the main reactant to determine basic reaction parameters. Figure 3 shows the progress of conversion to epoxide at different reaction temperatures and gives model fit as solid lines. Recalculated to yield, the yield at 40 min of reaction performed at 60°C is slightly higher than that at 50°C, whereas the yield in the reaction performed at 80°C is approximately twice higher than that at 50°C. The selectivity of the reactions is above 80%. The fastest reaction was at 80°C and, therefore, this temperature was taken as standard for the investigation of the effect of the catalyst composition, Table 1. In the second and third reactions the amount of hydrogen peroxide was reduced. In the fourth reaction both hydrogen peroxide and the amount of ortho‐phosphoric acid were reduced. The results are shown in Fig. 4. The initial catalyst composition gives the fastest reaction. Reaction rate is limited by the amount of hydrogen peroxide. The best molar equivalent is 57 with respect to the ortho‐phosphoric acid concentration.

#### *Flow epoxidation*

**Figure 5:**
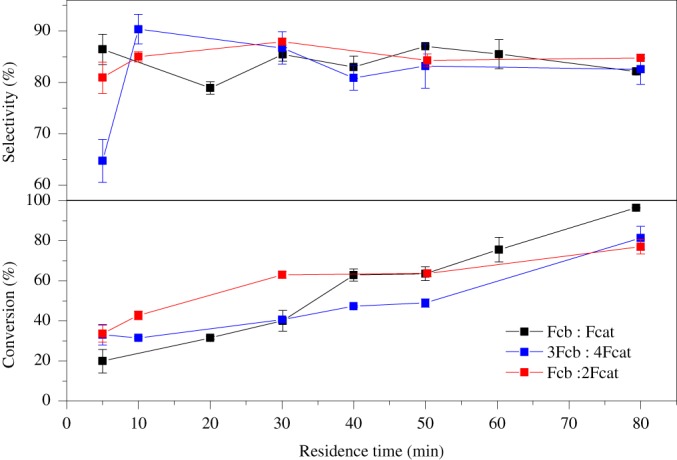
Influence of the ratio of flow rates of reactant to catalyst on the conversion of cocoa butter and selectivity to epoxide.

The influence of the ratio of the flow rates of the reactant to the catalyst solution was investigated. The ratio was varied between Fcb:Fcat = 1:1, 1:2, 3:4, see Figure 5. Variation in the flow rates affects the relative sizes of the organic and the aqueous phases slugs under Taylor flow regime. At short residence times, the increase in the amount of catalyst phase resulted in higher conversions, compared with 1:1 ratio of the flow rates. This is attributed to improved mass transfer. Better convection occurs within each slug and molecular diffusion occurs through the interfacial regions between the adjacent slugs. However, at longer residence times, the higher ratio of the aqueous phase produced lower conversions of cocoa butter in comparison with the 1:1 ratio of the flow rates. In this case, convection is slower and thus, migration of the active part of the catalyst is insufficiently fast to produce higher conversions by the solutions of higher ratio of aqueous phase. Epoxidation is limited by the rate of phase transfer of the catalyst from aqueous phase to the organic phase. At longer residence times the reaction with the lower amount of aqueous phase results in higher conversion.

#### *Comparison of epoxidation under batch and flow conditions*

Reactions under batch and flow conditions represent completely different reaction environments. First, reactions under batch conditions were performed solventless, which resulted in higher viscosity. The concentration profiles of reactants under batch and flow conditions are different. This may potentially affect selectivity, especially in the case of consecutive reactions, such as epoxidation, where ring‐opening may follow the formation of an epoxide. In the case of the phase transfer catalysis, reaction is biphasic and is dependent on high interfacial area, created by emulsification in a batch, or by a Taylor flow regime in a continuous flow processes, respectively. Thus, the influence of interfacial surface area and viscosity on the reaction selectivity and conversion should be investigated.

**Figure 6:**
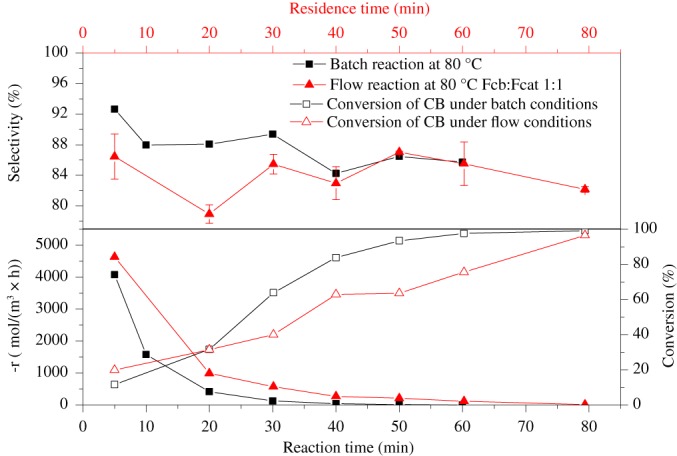
Dependence of the rate of disappearance of double bond and selectivity of epoxide on residence time (in the flow system) or reaction time (in the batch reactor).

Figure 6 shows a comparison of the rate of double bond conversion as a function of time and residence time for the two reactor systems. The rate of reaction is higher in the case of the flow system, despite the lower reactants concentration due to dilution. This results in the overall higher space‐time‐yield (STY): 327 and 295 mol m^−3^ h^−1^ for the flow and the batch reactors, respectively.

In contrast, the selectivity to epoxide decreases with increase in residence time and is always higher in batch reaction. This is attributed to the degradation of hydrogen peroxide in the flow reactor system, which results in higher water concentration and the increase in concentration of ortho‐phosphoric acid, which is a catalyst for diol formation.^27^ To confirm this we measured concentration of H_2_O_2_ as a function of time in the feed vessel with/without stirring and at the exit of the flow reactor. There was no observed decomposition of H_2_O_2_ in the feed vessel, whereas the residual concentration at the exit of the reactor was below the limit of detection by iodometric titration.

#### *Reaction calorimetry under batch conditions*

There is very little literature information on the heat of epoxidation reaction. For this reason we performed reaction calorimetry measurements to obtain this fundamental information, required for process development and optimisation.

The true heat flow for the reaction was equal to 3997 J and the heat flow for no reactive system was 1092 J. The enthalpy of reaction at 80°C can be obtained then from equation:

(26) $$\Delta H_{r}^{0}=\frac{\begin{array}{c} \left(\text{True heat flow in}\;a\;\text{reactive system}\right.\\ \left.-\text{True heat flow in the non reactive system}\right) \end{array}}{\left(\text{Number of moles of}\;\mathrm{CB}\;\text{in the reaction}\right)}$$

The enthalpy of epoxidation of cocoa butter was found to be mildly exothermic at −168 kJ mol^−1^. The enthalpy of epoxidation of soybean oil^26^ was reported to be −230 kJ mol^−1^, and from the limited data reported for acetic acid epoxidation^49^ with H_2_O_2_ the heat of reaction is estimated at −125.4 kJ mol^−1^. From NMR analysis of the reaction product obtained in the calorimeter, the starting cocoa butter reacted completely and the selectivity of reaction was 77%. This is below the typical selectivities obtained in batch (85%). The observed decrease was attributed to product decomposition in the case of the calorimeter experiment, when product composition was analysed after 18 h following the reaction. Therefore, we concluded that this observation does not affect the accuracy of the obtained heat of reaction value.

#### *Kinetic model*

In the multiphase reaction systems the effects of chemical reaction kinetics and mass transfer resistance are superimposed. This leads to complex estimation problems for which good initial guesses for the unknown parameters are needed to obtain useful results.^50^ To avoid these complications it is favourable to obtain separate measurements within the kinetically controlled and the mass transfer controlled regimes. Therefore, initial guesses were estimated with the data of those experiments indicating the least mass transfer or kinetic resistance, respectively. The first step was estimating initial guesses for the rate constants. The experiments with the highest stirrer speed were chosen because the least mass transfer resistance can be expected. Following the introduced approach of re‐parameterised Arrhenius equation, an isothermal fit for the experiments of 1000 and 1250 rpm stirrer speed at 80°C was performed and the result will be an initial guess for the reference rate constant for all experiments at 80°C.

Due to the absence of mass transfer resistance the measurements show pseudo‐first‐order behaviour with respect to the organic phase reaction. The concentration of active PTA–catalyst in the organic phase is nearly constant due to excess of hydrogen peroxide. While this allows a good estimate of the rate constant in the organic phase, it permits reliable estimate of the reference rate constant in the aqueous phase. With the current model it is impossible to estimate the mass transfer coefficient for all experiments simultaneously, because it depends on the stirrer speed. Therefore, the volumetric overall mass transfer coefficient *Ka* must be expressed in terms of the known stirrer speed. Based on the initial estimate for the reference rate constants the volumetric mass transfer coefficient for all other experiments at 80°C and various stirrer speeds was estimated and the following correlation was used to express the volumetric overall mass transfer coefficient as a function of the stirrer speed:

(27) $$\mathrm{Ka}=A_{\mathrm{rpm}}\text{exp}\left(B_{\mathrm{rpm}}n\right)$$

Consequently, the model parameter is replaced by Equation (27) within the integral model and, therefore, *A*_*rpm*_ and *B*_*rpm*_ are added as additional parameters to the model.

With the reference rate constants and the mass transfer coefficient as a function of the stirrer speed, the activation energies *E*_*a* , *org*_ and *E*_*a* , *aq*_ were estimated based on the experiments at 80°C.

**Figure 7:**
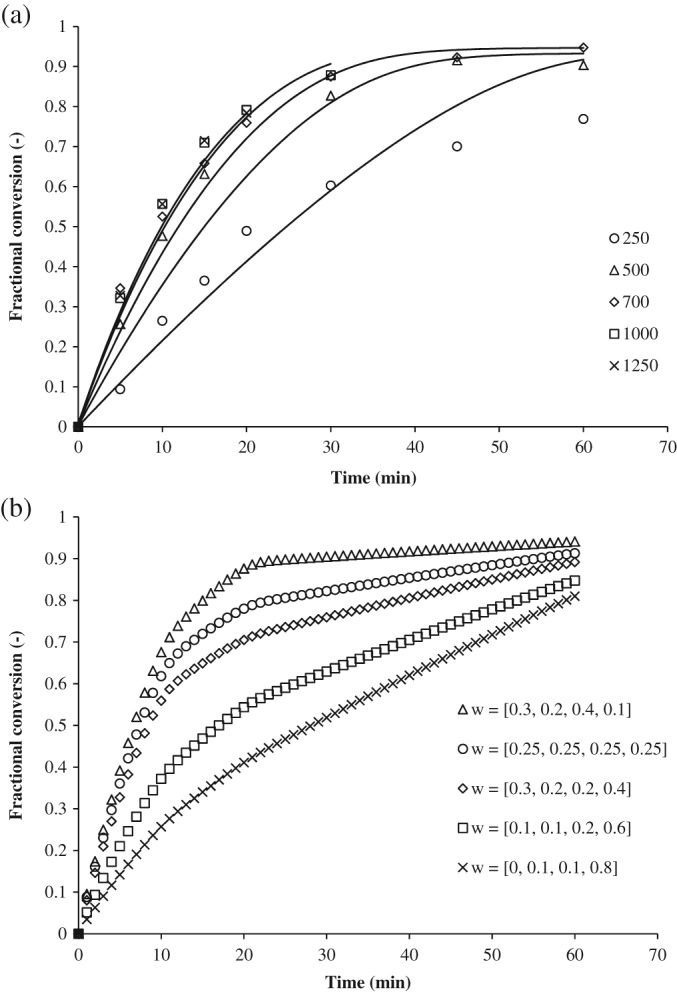
(A) Results of parameter estimation for different stirrer speeds (legend gives stirrer speed in rpm). (B) Effect of different droplet size distributions simulated with the differential model (legend gives the weight constant (w) from Equations (16) and (17)).

In the last step all the initial estimates were used as initial guesses to estimate all the parameters – *k*_*ref* , *org*_, *k*_*ref* , *aq*_, *E*_*a* , *org*_, *E*_*a* , *aq*_, *A*_*rpm*_ and *B*_*rpm*_ – simultaneously based on all the available experimental data to account for interdependencies. Figure 7(A) shows that the model predicts results for stirrer speeds 500–1250 rpm reasonably well, but the estimation fails for the experiment at 250 rpm. This may be due to the presence of a different droplet size distribution in the reactor at 250 rpm. Between 500 and 1250 rpm mono‐dispersed droplets can be assumed. Here the increase in the overall mass transfer coefficient stems partly from a decrease in droplet size and partly from the decreased mass transfer resistance at the interface due to the higher shear. However, at 250 rpm it is assumed that significantly larger droplets remain undispersed. The small droplets cause the behaviour as predicted by the model during the first 20 min, but thereafter the significantly lower overall reaction rate inside the bigger droplets causes the prediction to fail since the model assumes two ideally mixed bulk phases and cannot account for the effects of different droplet sizes.

This hypothesis is supported by simulations with the differential model (Equations (15) to (25)), if a droplet size distribution of large and small droplets is implemented. Figure 7(B) shows the conversion profile of three different droplet size distributions with the same kinetic constant. Although this is only a qualitative illustration of the model behaviour and not an actual fit of the experimental data, it clearly shows how the conversion curves bend, qualitatively representing the observed experimental behaviour. If a droplet size distribution could be obtained during the measurements, from videos or photographs, it seems likely that different flow patterns could be included into the differential model and the prediction would be considerably more accurate than with the integral model.

Excess hydrogen peroxide in most experiments permits a reliable estimate of the rate constant of the activation reaction in the aqueous phase. Although only a single experiment was done with hydrogen peroxide limitation, a satisfactory estimate was obtained and the model predicts the correct values for hydrogen peroxide limiting conditions, Figure 4.

Another important factor is the amount of catalyst which is available in its active state. It was assumed for the estimation, that the entire tungsten used in the experiment is present as PTA–catalyst. Figure 4 shows satisfactory prediction for a low tungsten concentration. The deviation of model and experiments at high conversions is likely to be associated with product decomposition, not accounted for in the model. Since different catalyst species could not be measured, no quantitative information could be obtained for how much of the added tungsten actually became an active PTA–catalyst species. To fully understand how different initial amounts of tungsten influence the reaction, if technically possible the different catalytic species should be measured in both the phases, to obtain a concentration profile and partition coefficients of the latter.

Besides these limitations and model failures for extreme values, the integral model predicts the measured data reasonably well and Table 3 shows that all parameters have physically meaningful values. The activation energy is far above 30 kJ mol^−1^ which can be considered as a lower level for activation energies. Table S1 (Supporting information) shows the 95% t‐value of each estimated parameter. The weighted residual value is less than the χ^2^ value thereby, indicating the satisfaction of χ^2^ test. The 95% confidence ellipsoids are shown in Fig. S1 (Supporting information). This test is based on whether the ellipsoidal area that represents the joint confidence region encloses the optimal point at its centre neighbourhood. However, the differential model at this stage can be used only for qualitative analysis of the reaction system, as it requires significantly more experimental detail, specifically the distribution of droplet sizes. If available, a differential model can be used for simulation of potential reactor options.

**Table 3:** Final estimated parameters for the integral model based on all cocoa butter experiments

Parameter	Final value
*k*_*ref* , *org*_ (*l*/*mol s*)	2.3721 (6.64 × 10^9^)^a^
*k*_*ref* , *aq*_ (*l*/*mol s*)	0.0713 (3.31 × 10^6^)^a^
*E*_*a* , *org*_ (*J*/*mol*)	63866.6
*E*_*a* , *aq*_ (*J*/*mol*)	51651.4
*A*_*rpm*_	0.0129
*B*_*rpm*_	0.0042

a Value in the parentheses represents the pre‐exponential factor of the reaction.

### Ring opening under batch conditions

Epoxides tend to undergo nucleophilic ring‐opening usually to form alcohol derivatives. These alcohol derivatives give rise to compounds that can be used as monomers in polyurethane synthesis.^51^, ^52^ In 2004 Zlatanić *et al*. synthesised six polyurethanes from polyols derived from canola, midoleic sunflower, soybean, linseed, sunflower and corn oils.^53^ The aim was to identify the effect different triglyceride structures had on the properties of any subsequent MDI derived polyurethanes. Initial ring‐opening was investigated using ortho‐phosphoric acid in water at 100°C for 24 h.^45^ This gave the desired polyol **3** in 93% yield (OH value 60 mg KOH g^−1^), accompanied by some hydrolysis (acid value 10 mg KOH g^−1^, *M*_w_ ∼1000 Da). Thermal analysis (DSC and TGA) of **3** indicated two melting points at 23 and 55°C and a T_10%_ decomposition point at 357°C with T_50%_ at 403°C.

Lewis acid mediated ring‐opening oligomerisation has been shown to provide oligomeric fatty acid or triglyceride polyols with higher molecular weights. The studies involving the use of 15% (mol) of BF_3_·Et_2_O as the catalyst without solvent at 20°C to yield oligomeric polyols **4**. Similar results were obtained with lower catalyst loading if hexane was used as solvent. Increasing the amount of Lewis acid increased the molecular weight with little effect on the polydispersity. Recent studies using BF_3_·Et_2_O in THF indicated that ring‐opening of vegetable oil epoxides with concurrent ring‐opening of THF could be achieved to give **5**.^54^ Ring‐opening with THF gave rise to higher molecular weight polyols with higher polydispersities.

Polyol (**3**) was characterised as : νmax / cm^−1^: 3341, 2915, 1736; ^1^H NMR (400 MHz, CDCl_3_) δ 5.26 (pent, *J* = 5.3 Hz, 1H), 4.22 (ddd, *J* = 17.6, 11.9, 5.3 Hz, 4H), 3.64 – 3.32 (m, 4H), 2.31 (t, *J* = 7.4 Hz), 1.64 – 1.56 (m, 6H), 1.53 – 1.43 (m, 4H), 1.37 – 1.23 (m, 70H), 0.88 (t, *J* = 6.3 Hz, 9H).; ^13^C NMR (101 MHz, CDCl_3_) δ 173.4, 172.9, 74.6, 74.6, 69.0, 62.2, 34.2, 34.1, 33.7, 33.6, 32.1, 32.0, 29.8, 29.7, 29.6, 29.5, 29.4, 29.2, 29.0, 25.8, 25.7, 24.9, 24.9, 22.8, 14.2.; ms (ES+) 917.8 [M+Na]^+^

The ring‐opening reactions in hexane were characterised by 400 MHz ^1^H NMR, (Fig. S2(A), Supporting information) which clearly showed the loss of epoxide protons at 2.92 ppm in **2** and the formation of protons adjacent to the alcohol functional groups between 3.30 and 3.60 ppm in **4**. MALDI‐TOF‐MS analysis (Fig. S2(B), Supporting information) clearly showed the repeating unit of the oligomers up to the tetramer. The peaks at 2371 and 1541 represent monomers where one ester has hydrolysed from the triglyceride to give a diglyceride derivative.

Characterisation of the polyols **5** obtained by ring‐opening in THF was also accomplished using ^1^H 400 MHz NMR and MALDI‐TOF‐MS. In this case the NMR shows protons characteristic of the formation of the grafted poly(THF) chain attached to the vegetable oil triglyceride at 3.4 and 1.6 ppm (Fig. S3(A), Supporting information). The MALDI‐TOF‐MS is more complicated than that for **4** (Fig. S3(B), Supporting information). In this case the repeating unit of ring‐opened THF can be seen (72 Da) superimposed onto triglyceride peaks (1115 Da corresponds with the Na adduct of a triglyceride with three THF units grafted, and 1919 Da corresponds to a dimeric triglyceride with two units of THF grafted). In addition, the ring‐opening can be terminated by hydrolysis (OH) or elimination (−18 Da, −H_2_O) and the fact that cocoa butter contains both C16 and C18 fatty acid chains means these series are also superimposed on ones differing by 29 Da (−C_2_H_5_). The properties of all the polyols are given in Table 4.

**Table 4:** Properties of the synthesised polyols

Entry	Solvent	Mol% BF_3_.Et_2_O	Yield polyol (%)	Gel permeation chromatography^a^		
				M_w_ (Da)	M_n_ (Da)	PDI
1	Bulk	15	56	6700	6100	1.10
2	hexane	3	19	6200	5700	1.09
3	hexane	5	49	6800	5900	1.15
4^b^	hexane	10	87	8400	7300	1.15
5	THF	5	82	36 800	16 000	2.30
6^b^	THF	10	93	84 000	30 000	2.80

a Measured using MMA as standard.

b These polyols were used to make PU4 and PU5.

### Polymerisation

**Figure 8:**
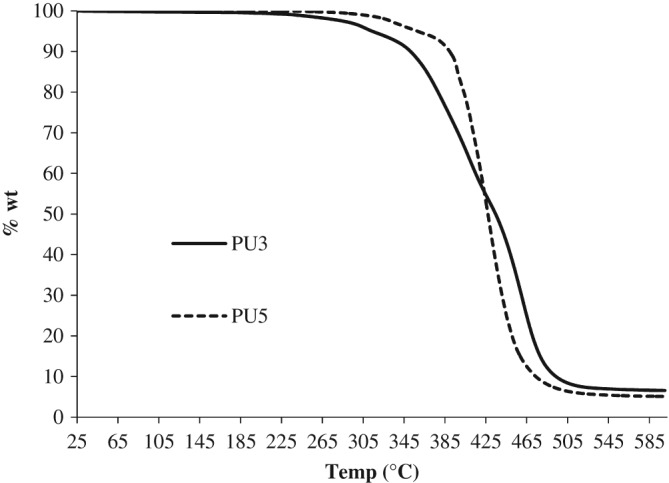
Thermal gravimetric analysis of PUs **3** and **5.**

As the majority of published work on polyurethanes derived from renewable vegetable oils has focused upon MDI based materials, we investigated the reaction between our monomers **3**–**5** and MDI.^55^, ^56^, ^57^, ^58^ A ratio of 1.05:1.00 (isocyanate:OH group) was used to ensure all hydroxyl groups reacted with any excess isocyanate reacting with the moisture in the air during the curing process. The PU prepared from polyol **4** was a viscous paste and no further characterisation data was obtained. Thermal analysis was carried out on PUs **3** and **5**, Figure 8. Vegetable oil based PUs generally show a two‐step degradation, with the first degradation occurring around 200°C, indicative of decomposition of the urethane linkages to amines and alkenes.^59^, ^60^ Both polymers **3** and **5** exhibited this behaviour with PU**3** showing lower thermal stability, see Table 5. Both PUs showed relatively low glass transition temperatures (PU3 = −15°C, PU5 = −75°C) which are substantially lower than those derived from sunflower *T*_g_ = 24 or soybean oil *T*_g_ = 31 °C.^61^ In addition, they exhibited lower tensile strength compared with sunflower oil (tensile strength = 14.8 MPa), and this is due to the lower hydroxyl values of the original monomers and, consequently, less cross‐linking in the PUs themselves.

**Table 5:** Properties of polyurethanes

Polyurethane	T_10%_(°C)	T_50%_(°C)	T_g_(°C)	Tensile strength(MPa)	Elongation at break(%)
PU3	353	435	−15	3.2	83
PU5	374	399	−75	5.9	610

## CONCLUSIONS

The process for conversion of waste cocoa butter into useful polyurethanes was established. Phase transfer catalyst was used for epoxidation of cocoa butter and the corresponding reaction parameters were identified by developing detailed kinetic model in batch mode. From the estimation results it can be concluded, that the rather simple integral model allows good predictions as long as the governing parameters of the liquid–liquid system such as temperature and droplet size distribution stay within a narrow range. Because the effects of the liquid–liquid system are all combined in a single parameter *Ka*, the integral model will be unreliable for the prediction of measurements from different flow‐regimes or temperature ranges. The differential model developed would be suitable for exploring a wider range of operating conditions, but at this stage lacks the required experimental detail.

Batch epoxidation was found to perform better than in flow owing to the decomposition of hydrogen peroxide. The ring opening of epoxide using tetrahydrofuran gives polyols with higher molecular weight and polydispersity with boron trifluoride diethyl etherate as catalyst. The polyols obtained can be successfully polymerised to obtain polyurethanes.

Thus, a complete pathway of utilisation of a bio‐waste feedstock from generation of a reactive intermediate to formation of end‐product polymers has been shown.

## Supporting information

Appendix S1.

## References

[1] Commission E , Green and Circular Economy, https://ec.europa.eu/jrc/en/research‐topic/green‐and‐resource‐efficient‐europe.

[2] Corma A , Iborra S and Velty A , Chemical routes for the transformation of biomass into chemicals. Chem Rev 107:2411–2502 (2007). 17535020 10.1021/cr050989d

[3] Lestari S , Mäki‐Arvela P , Beltramini J , Lu GQM and Murzin DY , Transforming triglycerides and fatty acids into biofuels. ChemSusChem 2:1109–1119 (2009). 19862784 10.1002/cssc.200900107

[4] Ozturk BO , Topoglu B and Sehitoglu SK , Metathesis reactions of rapeseed oil‐derived fatty acid methyl esters induced by monometallic and homobimetallic ruthenium complexes. Eur J Lipid Sci Technol 117:200–208 (2015).

[5] Campanella A , Fontanini C and Baltanas MA , High yield epoxidation of fatty acid methyl esters with performic acid generated *in situ* . Chem Eng J 144:466–475 (2008).

[6] Wang RP , Schuman T , Vuppalapati RR and Chandrashekhara K , Fabrication of bio‐based epoxy‐clay nanocomposites. Green Chem 16:1871–1882 (2014).

[7] Miao SD , Wang P , Su ZG and Zhang SP , Vegetable‐oil‐based polymers as future polymeric biomaterials. Acta Biomater 10:1692–1704 (2014). 24012607 10.1016/j.actbio.2013.08.040

[8] Kubicka D and Kaluza L , Deoxygenation of vegetable oils over sulfided Ni, Mo and NiMo catalysts. Appl Catal A ‐ Gen 372:199–208 (2010).

[9] Morgan T , Grubb D , Santillan‐Jimenez E and Crocker M , Conversion of triglycerides to hydrocarbons over supported metal catalysts. Top Catal 53:820–829 (2010).

[10] Kubicka D , Bejblova M and Vlk J , Conversion of vegetable oils into hydrocarbons over CoMo/MCM‐41 catalysts. Top Catal 53:168–178 (2010).

[11] Darocha GN , Brodzki D and Djegamariadassou G , Formation of alkanes, alkylcycloalkanes and alkylbenzenes during the catalytic hydrocracking of vegetable‐oils. Fuel 72:543–549 (1993).

[12] Chiappero M , Do PTM , Crossley S , Lobban LL and Resasco DE , Direct conversion of triglycerides to olefins and paraffins over noble metal supported catalysts. Fuel 90:1155–1165 (2011).

[13] Patel J , Elaridi J , Jackson WR , Robinson AJ , Serelis AK and Such C , Cross‐metathesis of unsaturated natural oils with 2‐butene. High conversion and productive catalyst turnovers. Chem Commun 44:5546–5547 (2005). 10.1039/b511626k 16358058

[14] Nordin NAM , Yamin BM , Yarmo MA , Pardan K and Alimuniar AB , Metathesis of palm oil. J Mol Catal 65:163–172 (1991).

[15] Refvik MD , Larock RC and Tian Q , Ruthenium‐catalyzed metathesis of vegetable oils. J Am Oil Chemists Soc 76:93–98 (1999).

[16] Ozturk C and Kusefoglu SH , New Polymers from epoxidized soybean oil with pyridine derivatives. J Appl Pol Sci 121:2976–2984 (2011).

[17] Barrett LW , Sperling LH and Murphy CJ , Naturally functionalized triglyceride oils in interpenetrating polymer networks. J Am Oil Chem Soc 70:523–534 (1993).

[18] Desroches M , Escouvois M , Auvergne R , Caillol S and Boutevin B , From vegetable oils to polyurethanes: synthetic routes to polyols and main industrial products. Polym Rev 52:38–79 (2012).

[19] Cai CS , Dai HG , Chen RS , Su CX , Xu XY , Zhang S *et al*, Studies on the kinetics of in situ epoxidation of vegetable oils. Eu J Lipid Sci Technol 110:341–346 (2008).

[20] Poli E , Clacens JM , Barrault J and Pouilloux Y , Solvent‐free selective epoxidation of fatty esters over a tungsten‐based catalyst. Catal Today 140:19–22 (2009).

[21] De Torres M , Arends I , Mayoral JA , Pires E and Jimenez‐Oses G , A highly efficient, green and recoverable catalytic system for the epoxidation of fatty esters and biodiesel with H_2_O_2_ . Appl Catal A: Gen 425:91–96 (2012).

[22] Sun SD , Yang GL , Bi YL and Liang H , Enzymatic epoxidation of corn oil by perstearic acid. J Am Oil Chem Soc 88:1567–1571 (2011).

[23] Dinda S , Goud VV , Patwardhan AV and Pradhan NC , Selective epoxidation of natural triglycerides using acidic ion exchange resin as catalyst. Asia‐Pacific J Chem Eng 6:870–878 (2011).

[24] Dinda S , Ravisankar V and Puri P , Development of bio‐epoxide from Nahor (Mesua ferrea Linn) oil. J Taiwan Inst Chem Eng 65:399–404 (2016).

[25] Somidi AKR , Sharma RV and Dalai AK , Catalytic vicinal diacylation of epoxidized triglycerides in canola oil. J Am Oil Chem Soc 92:1365–1378 (2015).

[26] Kralisch D , Streckmann I , Ott D , Krtschil U , Santacesaria E , Di Serio M *et al*, Transfer of the epoxidation of soybean oil from batch to flow chemistry guided by cost and environmental issues. ChemSusChem 5:300–311 (2012). 22287262 10.1002/cssc.201100445

[27] Cortese B , de Croon M and Hessel V , High‐temperature epoxidation of soybean oil in flow‐speeding up elemental reactions wanted and unwanted. Ind Eng Chem Res 51:1680–1689 (2012).

[28] Torrente‐Murciano L , Bishopp SD , Fox D and Scott JL , Biphasic epoxidation reaction in the absence of surfactants ‐ integration of reaction and separation steps in microtubular reactors. ACS Sustain Chem Eng 4:3245–3249 (2016).

[29] Lapkin A and Plucinski PK , Engineering factors for efficient flow processes in chemical industries, in Chemical Reactions and Processes under Flow Conditions, ed by Luis SV and RSC Garcia‐Verdugo E., Cambridge, 1–43 (2010).

[30] McPake CB , Murray CB and Sandford G , Epoxidation of alkenes using HOF center dot MeCN by a continuous flow process. Tetrahedron Lett 50:1674–1676 (2009).

[31] Mello R , Alcalde‐Aragones A , Olmos A , Gonzalez‐Nunez ME and Asensio G , Epoxidation of olefins with a silica‐supported peracid in supercritical carbon dioxide under Flow conditions. J Org Chem 77:4706–4710 (2012). 22533505 10.1021/jo300532f

[32] He W , Fang Z , Tian QT , Shen WD and Guo K , Tandem, effective continuous flow process for the epoxidation of cyclohexene. Ind Eng Chem Res 55:1373–1379 (2016).

[33] Cullen CJ , Wootton RCR and Mello AJ , Alkene epoxidation with a polystyrene immobilised metal salen catalyst in a continuous‐flow microfluidic reactor. J Appl Phys 105:102007–102010 (2009).

[34] Vanoye L , Wang J , Pablos M , de Bellefon C and Favre‐Reguillon A , Epoxidation using molecular oxygen in flow: facts and questions on the mechanism of the Mukaiyama epoxidation. Catal Sci Technol 6:4724–4732 (2016).

[35] Schotten C , Plaza D , Manzini S , Nolan SP , Ley SV , Browne DL *et al*, Continuous flow metathesis for direct valorization of food waste: an example of cocoa butter triglyceride. ACS Sust Chem Eng 3:1453–1459 (2015). 10.1021/acssuschemeng.5b00397 PMC4547494 26322250

[36] Venturello C and Daloisio R , Quaternary ammonium tetrakis(diperoxotungsto)phosphates(3‐) as a new class of catalysts for efficient alkene epoxidation with hydrogen peroxide. J Org Chem 53:1553–1557 (1988).

[37] Csanyi LJ and Jaky K , Peroxo‐oxometallate formation under phase‐transfer conditions. J Mol Catal 61:75–84 (1990).

[38] Yadav GD and Pujari AA , Epoxidation of styrene to styrene oxide: synergism of heteropoly acid and phase‐transfer catalyst under Ishii‐Venturello mechanism. Org Process Res Dev 4:88–93 (2000).

[39] Venturello C , Alneri E and Ricci M , A new effective catalytic‐system for epoxidation of olefins by hydrogen peroxide under phase‐transfer conditions. J Org Chem 48:3831–3833 (1983).

[40] Yadav GD and Satoskar DV , Kinetics of epoxidation of alkyl esters of undecylenic acid: comparison of traditional routes vs. Ishii‐Venturello chemistry. J Am Oil Chem Soc 74:397–407 (1997).

[41] Duncan DC , Chambers RC , Hecht E and Hill CL , Mechanism and dynamics in the H_3_PW_12_O_40_ [H_3_PWO_12_O_40_]‐catalysed selective epoxidation of terminal olefins by H_2_O_2_ ‐ formation, reactivity, and stability of (PO4 WO(O‐2)(2) (4))(3‐) {PO_4_[WO(O_2_)_2_]_4_}^4^-. J Am Chem Soc 117:681–691 (1995).

[42] Kozhevnikov IV , Mulder GP , Steverink‐de Zoete MC and Oostwal MG , Epoxidation of oleic acid catalyzed by peroxo phosphotungstate in a two‐phase system. J Mol Catal A ‐ Chem 134:223–228 (1998).

[43] Clark A , Keene B and Hignett R , Low‐Cost Synthesis and Evaluation of Polymers Prepared From Oilseed Rape and Euphorbia Lagascae Oil. Warwick University, Coventry, 32 (2001).

[44] Sherringham JA , Clark AJ and Keene RT , New chemical feedstock from unsaturated oils. Lipid Technol 12:129–132 (2000).

[45] Coles SR , Barker G , Clark AJ , Kirwan K , Jacobs D , Makenji K and Pink D , Synthetic mimicking of plant oils and comparison with naturally grown products in polyurethane synthesis. Macromol Biosci 8:526–532 (2008). 18322912 10.1002/mabi.200700238

[46] Chemisens, CPA 202 Reaction calorimeter system (Product Information) (2004).

[47] Singh J , Reaction calorimetry for process development: recent advances. Process Safety Progress 16:43–49(1997).

[48] Barison A , da Silva CWP , Campos FR , Simonelli F , Lenz CA and Ferreira AG , A simple methodology for the determination of fatty acid composition in edible oils through ^1^H NMR spectroscopy. Magn Reson Chem 48:642–650 (2010). 20589730 10.1002/mrc.2629

[49] Leveneur S , Estel L and Crua C , Thermal risk assessment of vegetable oil epoxidation. J Therm Anal Calorim 122:795–804 (2015).

[50] Michalik C , Brendel M and Marquardt W , Incremental identification of fluid multi‐phase reaction systems. AIChE J 55:1009–1022 (2009).

[51] Guo A , Cho YJ and Petrovic ZS , Structure and properties of halogenated and nonhalogenated soy‐based polyols. J Polym Sci Part A: Polym Chem 38:3900–3910 (2000).

[52] Petrovic ZS , Guo A and Zhang W , Structure and properties of polyurethanes based on halogenated and nonhalogenated soy‐polyols. J Polym Sci Part A: Polym Chem 38:4062–4069 (2000).

[53] Zlatanic A , Lava C , Zhang W and Petrovic ZS , Effect of structure on properties of polyols and polyurethanes based on different vegetable oils. J Polym Sci Part B: Polym Phys 42:809–819 (2004).

[54] Clark AJ and Hoong SS , Copolymers of tetrahydrofuran and epoxidized vegetable oils: application to elastomeric polyurethanes. Polym Chem 5:3238–3244 (2014).

[55] Petrovic ZS , Guo A , Javni I , Cvetkovic I and Hong DP , Polyurethane networks from polyols obtained by hydroformylation of soybean oil. Polym Int 57:275–281 (2008).

[56] Petrovic ZS , Zhang W and Javni I , Structure and properties of polyurethanes prepared from triglyceride polyols by ozonolysis. Biomacromolecules 6:713–719 (2005). 15762634 10.1021/bm049451s

[57] Lligadas G , Ronda JC , Galia M and Cadiz V , Polyurethane networks from fatty‐acid‐based aromatic triols: synthesis and characterization. Biomacromolecules 8:1858–1864 (2007). 17472338 10.1021/bm070157k

[58] Dumont MJ , Kong XH and Narine SS , Polyurethanes from benzene polyols synthesized from vegetable oils: dependence of physical properties on structure. J Appl Polym Sci 117:3196–3203 (2010).

[59] Javni I , Petrovic ZS , Guo A and Fuller R , Thermal stability of polyurethanes based on vegetable oils. J Appl Polym Sci 77:1723–1734 (2000).

[60] Del Rio E , Galia M , Cadiz V , Lligadas G and Ronda JC , Polymerization of epoxidized vegetable oil derivatives: ionic‐coordinative polymerization of methylepoxyoleate. J Polym Sci Part A: Polym Chem 48:4995–5008 (2010).

[61] Hong J , Luo Q , Wan XM , Petrovic ZS and Shah BK , Biopolymers from vegetable oils via catalyst‐ and solvent‐free ‘click’ chemistry: effects of cross‐linking density. Biomacromolecules 13:261–266 (2012). 22148512 10.1021/bm201554x

