# Supplementary material for: Direct valorisation of waste cocoa butter triglycerides via catalytic epoxidation, ring‐opening and polymerisation
Source: J Chem Technol Biotechnol. 2017 May 24;92(9):2254–66. doi: 10.1002/jctb.5292 (PMC5575473; doi:10.1002/jctb.5292)
Supplement: Supplementary file 1 — Appendix S1. [file JCTB-92-2254-s001.docx]

***Supporting Information***

List:

1. Validation of model assumptions.
2. Parameter estimation results (Table S1).
3. Model confidence ellipsoids (Figure S1).
4. Polymer characterisation (Figures S2 and S3).
5. ***Validation of assumption of isothermal experimental conditions***

The isothermal estimation of a reference rate constant is only applicable if the experiments can be assumed to be isothermal during the time of the entire experiment. A quick estimation of an upper limit for the expected temperature rise justifies this assumption: The heat of reaction was measured to be 168 kJ/mol and the apparent rate constant which can be obtained from a logarithmic plot from the available data. With the initial – and therefore maximal – concentration of cocoa butter and catalyst, an upper limit for the heat generation can be calculated.

$$\dot{Q}_{max}=V_{R}k_{app}c_{CB,0}\Delta H_{r}=0.045\left( L \right)\times0.0015\left( \frac{1}{s} \right)\times0.96\left( \frac{mol}{L} \right)\times168\left( \frac{kJ}{mol} \right)=11 W$$

This energy has to be removed by a temperature difference between the heated flask and the liquid. If the flask wall is assumed to be at a constant temperature of 80 °C and a contacting area of a third of the flask surface (sphere with 5cm diameter), and a heat transfer coefficient for forced convection 700 W m^-2^ K^-1^ of the temperature rise can be calculated as follows.

$$Q_{max}=UA(T_{R}-T_{flask})$$

$$T_{R}=T_{wall}+\frac{Q_{max}}{U\frac{\pi}{4}D_{flask}^{2}}=80 \left( ℃ \right)+\frac{11 \left( W \right)}{700 \left( \frac{W}{m^{2}K} \right)\times\frac{\pi}{4}\times{0.05}^{2} \left( m^{2} \right)}=86℃$$

With a maximum temperature of 86 °C and additional effects such as heat removal over the liquid surface inside the stirred flask neglected, the use of an isothermal reference rate constant seems justified.

**Table S1:** Results of parameter estimation

| **Parameter** | **Optimal estimate** | **95%**  **confidence interval** | **95%**  **t-value** | **Std. deviation** | **Reference t-value** | **Weighted residual** | **χ2 value** |
| --- | --- | --- | --- | --- | --- | --- | --- |
| $A_{rpm}$ | 0.01294 | 0.00649 | 1.992 | 0.00328 | 1.654 | 169.751 | 177.39 |
| $B_{rpm}$ | 0.0042 | 0.0013 | 3.236 | 0.00065 |  |  |  |
| $k_{ref, aq} (l/{mol}s)$ | 0.0713 | 0.0159 | 4.473 | 0.00806 |  |  |  |
| $E_{a,aq} (J/{mol)}$ | 51651.1 | 26016.48 | 1.985 | 13165.74 |  |  |  |
| $k_{ref, org} (l/{mol}s)$ | 2.3721 | 0.812 | 2.92 | 0.411 |  |  |  |
| $E_{a,org} (J/{mol)}$ | 63866.6 | 28679.94 | 2.226 | 14513.6 |  |  |  |

(0.01294, 0.0042)

**(A)**

**(B)**

**(C)**

**Figure S1.** 95% confidence ellipsoids **(A)** Mass transfer coefficient parameters A and B **(B)** Frequency factor at reference temperature and activation energy of catalyst activation **(C)** Frequency factor at reference temperature and activation energy of epoxidation.

NMR of the epoxidised cocoa butter : ν_max_ / cm^-1^: 2914, 1729, 1209; ^1^H NMR (400 MHz, CDCl_3_): δ_H_ = 5.26 (1H, pent, *J* = 5.0 Hz), 4.22 (4H, ddd, *J* = 17.8, 11.9, 5.0 Hz), 3.00 – 2.82 (2H, m), 2.31 (6H, t, *J* = 7.5 Hz), 1.65 – 1.56 (6H, m), 1.51 – 1.46 (4H, m), 1.46 – 1.15 (72H, m), 0.88 (9 H, t, *J* = 6.5 Hz); ^13^C NMR (100 MHz, CDCl_3_): δ_C_ = 173.4, 172.9, 69.0, 62.2, 57.3, 57.2, 34.2, 34.1, 32.0, 31.9, 29.8, 29.8, 29.7, 29.6, 29.6, 29.5, 29.4, 29.3, 29.2, 29.1, 27.9, 26.7, 25.0, 24.9, 22.8, 14.2; *ms* (ES+) 889.6 [M+Na]^+^.

**(A)**

**(B)**

**Figure S2. (A)** ^1^H 400 MHz NMR of reaction to produce polyols **4** from ring opening of **2** **(B)** ^1^H MALDI-TOF-MS of polyol **4** from ring opening of **2** in hexane.

**(A)**

**(B)**

**Figure S3. (A)** ^1^H 400 MHz NMR of reaction to produce polyols **5** from ring opening of **2** **(B)** ^1^H MALDI-TOF-MS of polyol **5** from ring opening of **2** in THF.
